# Massive Recurrent Parotid Carcinoma in a 90-Year-Old Patient: A Case Report and Review of Management Strategies

**DOI:** 10.7759/cureus.103561

**Published:** 2026-02-13

**Authors:** Younes Banana, Ayman El Mahjoubi, Achraf Sbai, Drissia Benfadil, Azzedine Lachkar

**Affiliations:** 1 Department of Otolaryngology-Head and Neck Surgery, University Hospital Center Mohammed VI, Oujda, MAR

**Keywords:** carcinoma ex-pleomorphic adenoma, chemo-radiation, epithelial-myoepithelial carcinoma, parotid gland carcinoma, salivary gland carcinoma

## Abstract

Carcinoma ex pleomorphic adenoma is a rare malignant salivary gland tumor arising from the malignant transformation of a pre-existing pleomorphic adenoma and is associated with significant diagnostic and therapeutic challenges. We report the case of a 90-year-old male with a long-standing history of recurrent pleomorphic adenoma of the parotid gland, who presented with a rapidly enlarging parotid mass associated with facial nerve involvement and parapharyngeal extension. Histopathological examination confirmed the diagnosis of epithelial-myoepithelial carcinoma ex pleomorphic adenoma. This case highlights the clinical presentation, radiological findings, and histopathological features of this uncommon malignancy and adds to the existing literature by emphasizing the diagnostic complexity and management considerations of carcinoma ex pleomorphic adenoma in elderly patients.

## Introduction

Salivary gland tumors represent a heterogeneous and histologically diverse group of neoplasms, encompassing both benign and malignant pathologies. The parotid gland is the most frequently affected site, accounting for approximately 70% of all salivary gland tumors. Among these, the majority are benign lesions, with pleomorphic adenoma being the most common. However, a significant diagnostic and therapeutic challenge arises when a benign pleomorphic adenoma undergoes malignant transformation, resulting in carcinoma ex pleomorphic adenoma (Ca ex PA), a rare but aggressive malignancy [1].

Ca ex PA typically arises after a long-standing history of untreated or recurrent pleomorphic adenoma, often evolving silently over several years. Clinical presentation may remain insidious until the lesion reaches an advanced stage, by which time invasion of adjacent structures or regional metastasis may have already occurred. This late presentation contributes to the often poor prognosis and underscores the importance of long-term surveillance of pleomorphic adenomas following excision [1].

Histopathologically, Ca ex PA encompasses a range of malignant subtypes, including adenocarcinoma not otherwise specified, salivary duct carcinoma, and epithelial-myoepithelial carcinoma (EMC). The epithelial-myoepithelial subtype is particularly rare and poses diagnostic challenges, as it is characterized by a biphasic architecture composed of inner ductal epithelial cells and an outer layer of clear myoepithelial cells. Although this pattern can be distinctive, it may be subtle or difficult to recognize in poorly differentiated tumors or in cases with extensive infiltration. From a clinical and biological standpoint, EMC generally exhibits a less aggressive course than high-grade salivary gland malignancies such as salivary duct carcinoma; however, its behavior remains unpredictable, with a recognized risk of local recurrence and distant metastasis. This intermediate biological profile has important implications for prognosis and long-term management [2].

Management of Ca ex PA requires a multidisciplinary approach integrating surgery, radiotherapy, and, in selected cases, systemic chemotherapy. However, therapeutic decisions are often complicated by the patient's age, comorbidities, and the tumor’s extent at diagnosis. Early recognition and histopathologic classification are essential for optimizing outcomes. Despite advances in imaging and surgical techniques, the prognosis for patients with Ca ex PA, particularly in advanced stages or in elderly individuals, remains guarded [3].

In this report, we present the case of a 90-year-old male with a long-standing history of pleomorphic adenoma of the parotid gland, previously managed with multiple resections, who subsequently developed a recurrent mass. Histopathological analysis confirmed the diagnosis of epithelial-myoepithelial Ca ex PA. This case is documented to contribute to the limited body of literature on epithelial-myoepithelial Ca ex PA by emphasizing its diagnostic complexity and therapeutic challenges, particularly in elderly patients.

## Case presentation

A 90-year-old male with a long-standing history of pleomorphic adenoma of the left parotid gland was referred to our department for evaluation of a large parotid mass. The patient was diagnosed in January 2016 with a pleomorphic adenoma following an exofacial parotidectomy performed for a parotid mass that had been progressively evolving for approximately five months prior to surgery. Despite surgery, the tumor recurred, leading to a second resection in February 2018, followed by a third surgical excision in August 2022, each time for recurrent disease.

In February 2025, the patient presented with a new left parotid mass, which, according to his account, had been progressively enlarging for approximately one year. The mass initially appeared as a painless swelling but subsequently demonstrated rapid growth. Shortly after the reappearance of the mass, the patient developed a facial palsy that progressed gradually over time.

**Figure 1:**
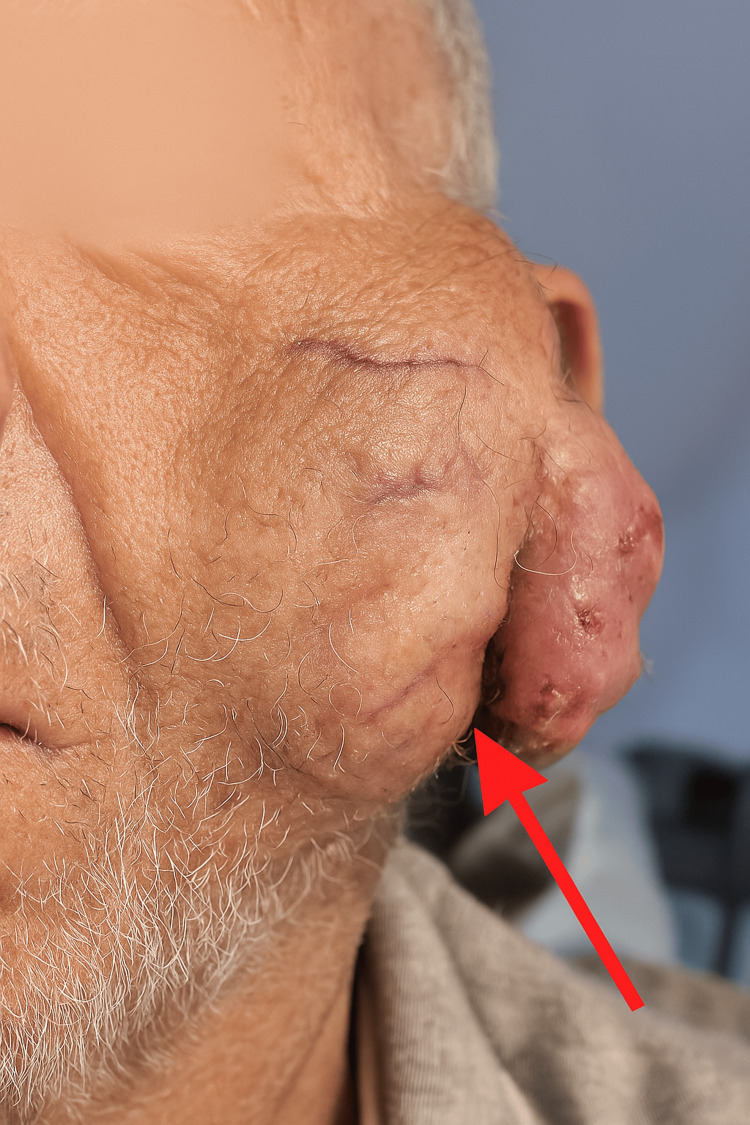
Clinical image showing a large left parotid mass with ulceration and prominent superficial venous network. The red arrowhead indicates the central area of tumor ulceration.

On admission, clinical examination revealed a large, hard, and multilobulated mass measuring more than 15 cm in its greatest dimension, occupying the left parotid region (Figure 1). The overlying skin was markedly thinned and ulcerated in several areas, reflecting local tumor aggression and pressure-related ischemic changes. The mass was immobile, firmly adherent to deep planes, and tender on palpation. Ipsilateral cervical lymphadenopathy at level IIb, measuring approximately 3 cm, was palpable, suggesting regional lymphatic involvement. Neurological examination identified a House-Brackmann grade II facial palsy. Endobuccal examination demonstrated medial displacement of the lateral pharyngeal wall, with a firm bulging mass palpable in the parapharyngeal space, consistent with deep tumor extension.

**Figure 2:**
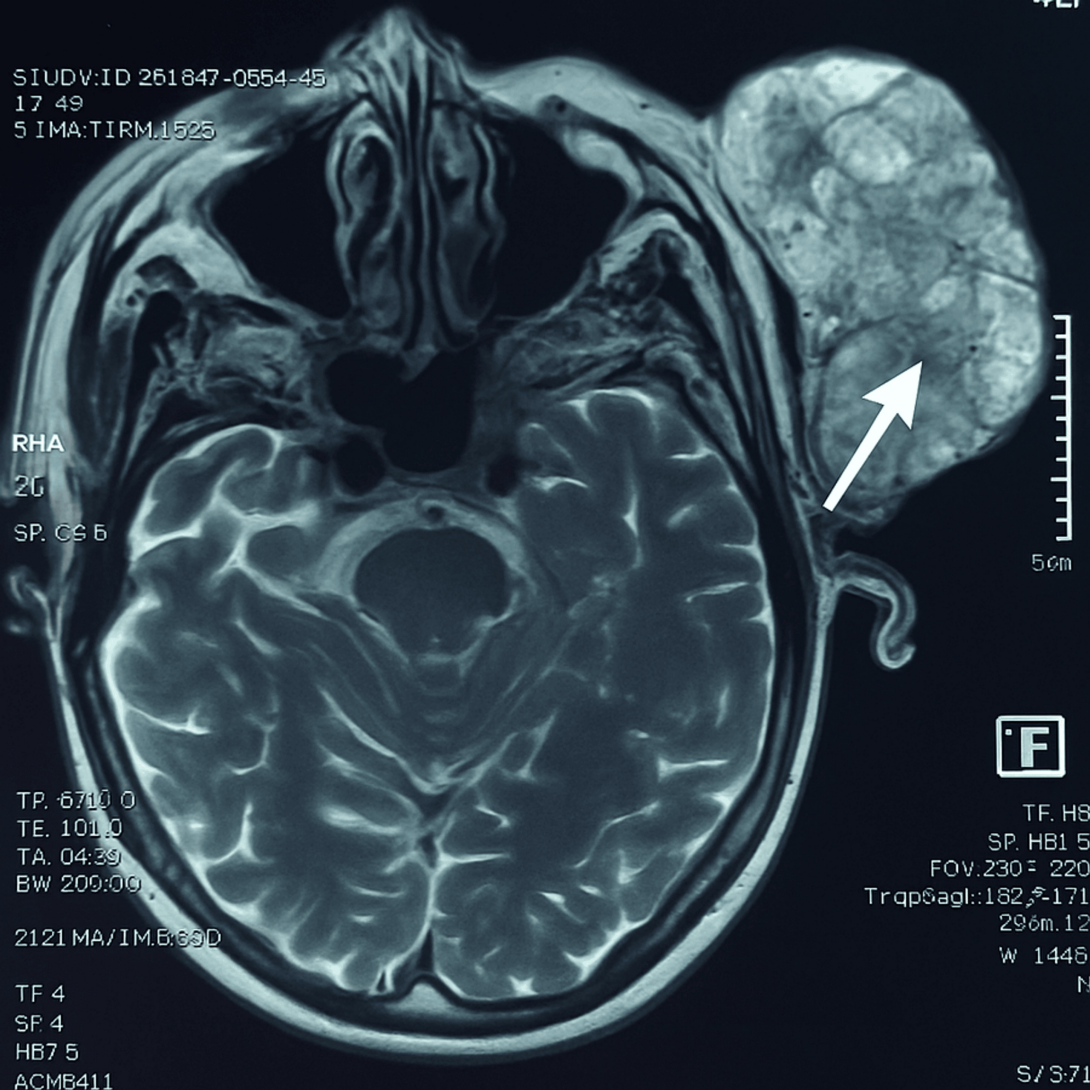
Axial T2-weighted MRI scan showing a large heterogeneous parotid mass occupying the left parotid region, consistent with locally advanced carcinoma.

MRI was performed to evaluate the extent of local invasion. Axial T2-weighted sequences (Figure 2) showed an expansive lesion with irregular margins and heterogeneous signal intensity. The tumor was in close contact with the masseter muscle, the ascending mandibular ramus, and extended into the retro- and pre-styloid spaces. The apparent diffusion coefficient (ADC) measured at 1.06 was suggestive of a low- to intermediate-grade malignancy. A contrast-enhanced cervico-thoracic CT ruled out distant metastases but confirmed the persistence of ipsilateral level IIb lymphadenopathy.

**Figure 3:**
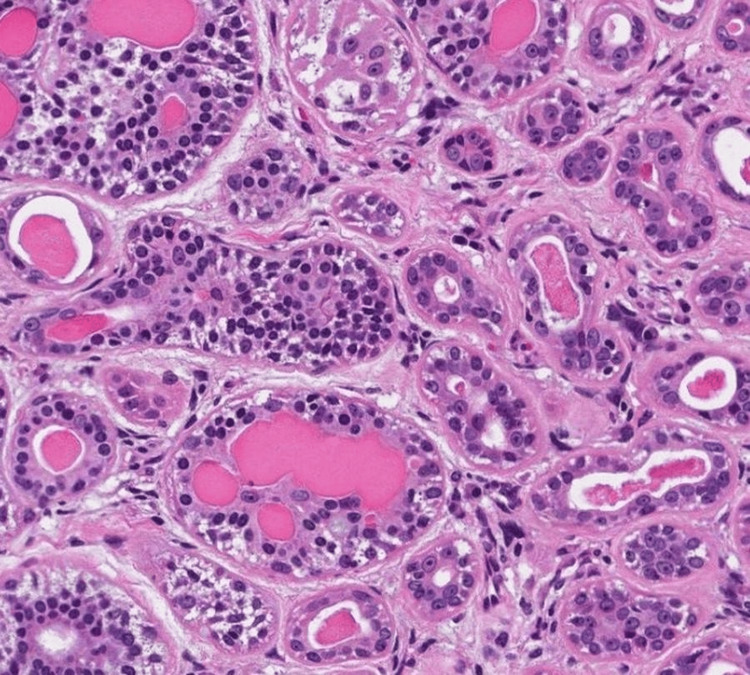
Histological image showing an epithelial-myoepithelial carcinoma under the microscope. This image shows a tumor composed of round tubules with an inner layer of ductal epithelial cells and an outer layer of myoepithelial cells.

A biopsy of the mass was performed. Histological analysis confirmed the diagnosis of EMC arising from a pleomorphic adenoma. The lesion exhibited features of malignancy without perineural or lymphovascular invasion (Figure 3).

Given the tumor’s advanced stage (T4b N1 M0), its extensive local invasion, and the patient’s age and comorbidities, the multidisciplinary tumor board concluded that surgery was contraindicated. A palliative-intent systemic chemotherapy regimen was therefore initiated with the goal of tumor burden reduction, symptom control, and potential reassessment for surgery.

The patient received cisplatin 75 mg/m² on Day 1 and 5-fluorouracil 1000 mg/m²/day as continuous infusion for four days. Given the patient's frailty, dosages were reduced by 20% from standard protocols. After two cycles, MRI showed 25% reduction in tumor volume, with improved ulceration and stable lymphadenopathy. Facial palsy remained unchanged. Toxicity was limited to grade 1-2 nausea and fatigue, with preserved renal and hematologic function.

After four cycles, MRI showed disease stabilization. The mass remained fixed to deep structures, making surgery still unfeasible. The patient was thus referred for palliative radiotherapy to control local progression and avoid complications such as hemorrhage or superinfection. He remains under regular follow-up with palliative support.

## Discussion

EMC ex pleomorphic adenoma is a rare salivary gland malignancy, predominantly affecting elderly individuals and most commonly originating in the parotid gland [4]. Similar to cases reported in the literature, malignant transformation in our patient occurred after a long-standing history of pleomorphic adenoma with multiple recurrences, a well-established risk factor for Ca ex PA. Several published series emphasize that repeated recurrences and prolonged tumor evolution significantly increase the risk of malignant transformation, which is consistent with the clinical course observed in our case [5].

Clinically, our patient presented with features frequently described in advanced EMC cases, including rapid tumor enlargement, facial nerve palsy, skin ulceration, and cervical lymphadenopathy. These signs are widely recognized as indicators of malignant transformation rather than benign recurrence in patients with a history of pleomorphic adenoma. However, compared with some reported cases in which facial palsy appears abruptly and is associated with perineural invasion, the facial nerve dysfunction in our patient developed progressively and without histological evidence of perineural spread. This discrepancy illustrates the variability between clinical presentation and histopathological findings in EMC [6].

Histologically, EMC is characterized by a distinctive biphasic architecture composed of inner epithelial and outer myoepithelial cells, a hallmark feature that was clearly identified in our case. While the literature describes histologic variants with oncocytic or squamous differentiation that may be associated with more aggressive behavior, such features were not observed in our patient [7]. Despite the absence of overtly aggressive histopathological characteristics, the tumor exhibited extensive local invasion, highlighting the well-documented discordance between histologic grade and clinical aggressiveness in EMC ex pleomorphic adenoma.

Although EMC is generally classified as a low- to intermediate-grade malignancy, its biological behavior remains unpredictable. Local recurrence, nodal involvement, and distant metastases have been reported, particularly in advanced-stage tumors or when optimal surgical management is not feasible. A population-based study by Gore reported a five-year overall survival rate of approximately 72%, underscoring the prognostic impact of tumor stage and completeness of resection [8]. In contrast to cases treated with curative surgical intent, our patient presented with advanced disease (T4b N1 M0), rendering surgical intervention contraindicated, a scenario described in a limited but clinically relevant subset of reported cases.

Due to the rarity of EMC ex pleomorphic adenoma, standardized chemotherapy protocols are lacking. Nevertheless, platinum-based regimens remain the most frequently used systemic treatments in unresectable or metastatic salivary gland carcinomas [9]. In our case, the combination of cisplatin and continuous-infusion 5-fluorouracil resulted in partial tumor regression and subsequent disease stabilization. These findings are consistent with previously published case-based experiences reporting modest but meaningful responses to similar regimens in advanced salivary gland malignancies [9].

Radiotherapy also plays an important role in the palliative management of locally advanced EMC. Several reports support its use for symptom control, improved local disease stabilization, and prevention of complications such as bleeding or superinfection in ulcerated tumors. In our patient, radiotherapy was implemented following chemotherapy-induced stabilization, aligning with approaches described in the literature for unresectable EMC and contributing to maintenance of local control and patient comfort [10].

Given the rarity and heterogeneity of EMC ex pleomorphic adenoma, management must be individualized and guided by a multidisciplinary tumor board. In elderly or frail patients, as illustrated in this case, balancing oncologic control with quality-of-life considerations is essential. Long-term follow-up remains critical due to the potential for delayed recurrence or metastatic spread, even in tumors with relatively indolent histological features.

## Conclusions

This case highlights the clinical complexity of giant parotid carcinoma arising from the malignant transformation of a recurrent pleomorphic adenoma in an elderly patient. Long-standing disease with multiple recurrences led to advanced local invasion and facial nerve involvement, ultimately precluding surgical management. In this context, a multidisciplinary, palliative-oriented approach allowed partial tumor control and symptom stabilization, underscoring the challenges of managing advanced salivary gland malignancies in frail patients.
